# Reexamining the Validity and Reliability of the Clinical Version of the Iowa Gambling Task: Evidence from a Normal Subject Group

**DOI:** 10.3389/fpsyg.2013.00220

**Published:** 2013-05-29

**Authors:** Ching-Hung Lin, Tzu-Jiun Song, Ying-Ying Chen, We-Kang Lee, Yao-Chu Chiu

**Affiliations:** ^1^Department of Psychology, Soochow UniversityTaipei, Taiwan; ^2^Brain Research Center, National Yang-Ming UniversityTaipei, Taiwan; ^3^Biomedical Electronics Translational Research Center, National Chiao Tung UniversityHsinchu, Taiwan; ^4^Biomedical Engineering R&D Center, China Medical UniversityTaichung, Taiwan; ^5^Department of Art Psychotherapy, The School of Health and Social Sciences, Queen Margaret UniversityEdinburgh, UK

**Keywords:** Iowa gambling task, clinical Iowa gambling task version, prominent deck B phenomenon, gain-loss frequency, expected value, validity, reliability

## Abstract

Over past decade, the Iowa gambling task (IGT) has been utilized to test various decision deficits induced by neurological damage or psychiatric disorders. The IGT has recently been standardized for identifying 13 different neuropsychological disorders. Neuropsychological patients choose bad decks frequently, and normal subjects prefer good expected value (EV) decks. However, the IGT has several validity and reliability problems. Some research groups have pointed out that the validity of IGT is influenced by the personality and emotional state of subjects. Additionally, several other studies have proposed that the “prominent deck B phenomenon” (PDB phenomenon) – that is, normal subjects preferring bad deck B – may be the most serious problem confronting IGT validity. Specifically, deck B offers a high frequency of gains but negative EV. In the standard IGT administration, choice behavior can be understood with reference to gain-loss frequency (GLF) rather than inferred future consequences (EV, the basic assumption of IGT). Furthermore, using two different criteria (basic assumption vs. professional norm) results in significantly different classification results. Therefore, we recruited 72 normal subjects to test the validity and reliability of IGT. Each subject performed three runs of the computer-based clinical IGT version. The PDB phenomenon has been observed to a significant degree in the first and second stages of the clinical IGT version. Obviously, validity, reliability, and the practice effect were unstable between two given stages. The present form of the clinical IGT version has only one stage, so its use should be reconsidered for examining normal decision makers; results from patient groups must also be interpreted with great care. GLF could be the main factor to be considered in establishing the constructional validity and reliability of the clinical IGT version.

## Introduction

Designed in 1994, the Iowa gambling task (IGT) has become one of the most complicated tasks used to study executive functions and emotionally driven decision making under uncertainty (Bechara et al., 1994, 1997, 1998, 1999, 2000). Original studies have shown that patients with ventromedial prefrontal cortex (VMPFC) lesions (Damasio et al., 1991; Bechara et al., 1994, 1997, 1998, 1999, 2000) and emotion system deficits (Bechara, 2001, 2003, 2004; Bechara and Damasio, 2002, 2005; Bechara et al., 2002) perform less well in IGT than do normal decision makers (Damasio, 1994).

The IGT was originally designed to test decision-making ability and frontal cortex functioning in highly ambiguous circumstances, with the goal of simulating real-life choice behavior (Eslinger and Damasio, 1985; Damasio et al., 1991; Damasio, 1994). Subjects are asked to play the IGT where they must intuitively determine the internal rules of the game. They are told that the game may end at any moment and that they must win as much as possible or lose as little as possible. Hence, subjects initially have no idea of the game’s internal structure. In all IGT versions (Tables 1 and 2), four decks are used and the endpoint is the 100th trial. Decks A and B are disadvantageous decks, with 10 cards in each deck having a negative expected value (EV). Conversely, decks C and D are advantageous decks, with 10 cards in each deck having a positive expected return. The basic assumption is that subjects significantly preferring the bad decks over the good ones are myopic decision makers who are insensitive to the bad long-term outcome offered by these decks (Bechara et al., 1994, 1997, 1998, 1999, 2000).

**Table 1:** **The gain-loss structure of original version in Iowa gambling task (Bechara et al., 1994)**.

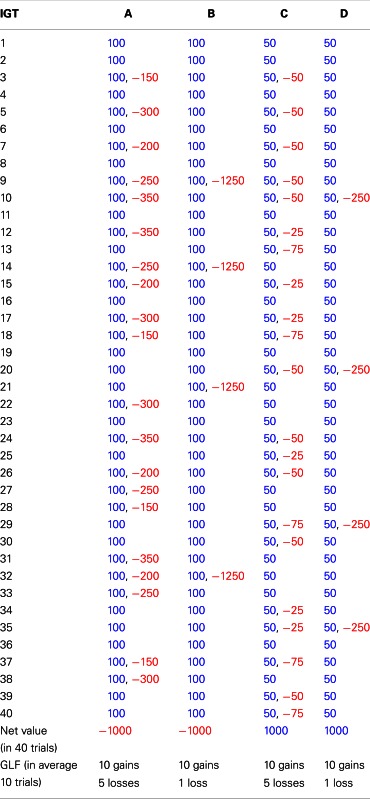

*The red and blue represented the loss and gain respectively*.

**Table 2:** **The gain-loss structure of clinical version in Iowa gambling task (Bechara, 2007)**.

IGT	A	B	C	D
Gain in each trial	80∼170	80∼170	40∼95	40∼95
1				
2				
3	−150		−50	
4				
5	−300		−50	
6				
7	−200		−50	
8				
9	−250	−1250	−50	
10	−350		−50	−250
11				
12	−350		−25	
13			−75	
14	−250	−1500		
15	−200		−25	
16				
17	−300		−25	
18	−150		−75	
19	−250			
20			−50	−275
21	−250	−1750		
22	−300		−25	
23				
24	−350		−50	
25			−25	
26	−200		−50	
27	−250			
28	−150		−25	
29	−250		−75	−300
30			−50	
31	−350		−25	
32	−200	−2000		
33	−250		−25	
34	−250		−25	
35	−150		−25	−325
36				
37	−150		−75	
38	−300		−25	
39	−350		−50	
40			−75	
41	−350		−25	
42	−200			
43	−250		−25	
44	−250		−25	
45	−150		−25	−350
46		−2250	−25	
47	−150		−75	
48	−300		−25	
49	−350		−50	
50	−250		−75	
51	−350		−25	
52	−200		−25	
53	−250		−25	
54	−250		−25	
55	−150		−25	
56	−250		−25	
57	−150		−75	
58	−300	−2500	−25	−375
59	−350		−50	
60	−250		−75	
EV (in 60 trials)	−3750	−3750	1875	1875
GLF (in average 10 trials)	10 gains	10 gains	10 gains	10 gains
	5∼10 losses	1 loss	5∼10 losses	1 loss

Over the last few years, various IGT versions have been generated (Bechara et al., 1994, 2000; Bechara, 2007). Most versions have confirmed the validity of the basic assumption that the emotional system of normal decision makers enables them to foresee the long-term benefits (EV) of particular choices. The Iowa group has published a clinical IGT version for use in quantitative assessment, and this version’s evaluative validity has been claimed for 13 neurological and psychiatric disorders, including focal brain lesions, intact older adults, substance addiction, pathological gambling, schizophrenia, obsessive-compulsive disorder, anorexia nervosa, obesity, chronic pain, ADHD, aggression disorders, affective disorders, and Huntington’s disease (Bechara, 2007).

The original IGT has been modified to develop a new computer-based clinical IGT version and further confirm the decision dysfunction of VMPFC patients (Bechara et al., 2000; Bechara, 2007). This clinical IGT version (Table 2) is different from the original version (Bechara et al., 1994) in many ways:

1. In the clinical IGT version, various rewards are given in each trial. In the original IGT version, the reward in each trial for decks A and B is $100, whereas the reward in each trial for decks A and B in the clinical IGT version is $100 on average. Similar adjustments are true for decks C and D.
2. The average immediate reward for decks A and B in each set of 10 trials is increased by $10 in the clinical IGT version and that for decks C and D is increased by $5.
3. For decks A and C in the clinical IGT version, the number of punishments is gradually increased in each set of 10 trials and the value of each punishment is kept at the same magnitude. Conversely, for decks B and D, the number of punishments is kept the same in each set of 10 trials and the value of each punishment is gradually increased. For instance, deck A (C) in the first set of 10 trials has “five” punishments, and the value ranges from $150 to $350 ($25 to $75). The second set of 10 trials has “six” punishments, and the value also ranges from $150 to $350 ($25 to $75). In contrast, deck B (D) in the first set of 10 trials has only one punishment with a value of $1250 ($250). The second set of 10 trials also has only one punishment with a value of $1500 ($275).
4. For an average of 10 trials (one set), decks A and B in the clinical IGT version have negative EV, and such negative EV is increased by $150 in each set (10 trials). Conversely, decks C and D have positive EV for an average of 10 trials (one set), and such positive EV is increased by $25 in each set (10 trials).

The foregoing modifications enhance EV contrast between the advantageous and disadvantageous decks; bad decks A and B become worse and good decks C and D become better in the long run (Bechara et al., 2000; Bechara, 2007). Bechara et al. (2000) demonstrated that the results of the original and clinical IGT versions for normal and VMPFC groups do not differ significantly. Moreover, they combined the computer-based and inverted IGT versions to illustrate that the dysfunction associated with VMPFC is insensitivity to future consequence (for a detailed description, see Bechara et al., 2000).

Based on the computer-based clinical IGT version, Bechara (2007) published a professional manual for clinical assessment. However, prior to the publication of the IGT manual, few studies had already examined the new version’s constructional validity. To examine the constructional validity of IGT, Buelow and Suhr (2009) reviewed numerous studies related to executive functions, decision making, and IGT, including studies on brain damage, functional imaging, children and adolescents, adults, drug abusers, pathological gamblers, OCD, and schizophrenia. They also discussed three issues that may influence the constructional validity of IGT: (1) the kind of decision abilities measured by IGT; (2) the absence of evidence of IGT reliability; and (3) the impact of personality traits and emotional state on IGT performance. They demonstrated that a small part of normal control groups perform worse in IGT and interpreted this finding based on two factors, namely, personality and emotional state. Suhr and Tsanadis (2007) suggested that bad IGT performance is related to high scores of reward responsiveness, fun seeking, and negative emotional states. However, they provided inconsistent evidence in their subsequent study (Hammers and Suhr, 2010). Furthermore, Horstmann et al. (2012) have also demonstrated that there exist various decision patterns in a healthy subject group. In short, reliability and validity are still critical problems in the clinical IGT version.

Over the past decade, an increasing number of investigations have pointed out a serious design flaw and a confounding factor in the IGT. Gain-loss frequency (GLF), rather than EV, can predict the choice behavior of the control group of normal decision makers (Wilder et al., 1998; Fernie and Tunney, 2006; Chiu and Lin, 2007; Fernie, 2007; Lin et al., 2007, 2008, 2009; Chiu et al., 2008; Fum et al., 2008; Stocco and Fum, 2008; Stocco et al., 2009; Napoli and Fum, 2010). This “prominent deck B phenomenon” (PDB phenomenon) refers to the finding that normal subjects prefer bad deck B, which offers high-frequency gain but has a negative EV (Lin et al., 2007; Chiu et al., 2012). Researchers have suggested that under uncertainty, even normal decision makers are most influenced by GLF; they are driven by immediate prospects rather than by long-term benefits. Foresighted decision making is very difficult in highly ambiguous situations. This viewpoint of immediate gain-loss is consistent with the behavioral decision literature, which describes the prospect theory (Kahneman and Tversky, 1979; Kahneman, 2003).

Growing research on clinical populations and behavioral modeling has revealed that GLF is the primary variable affecting the choices not only of control groups (Ahn et al., 2008; Horstmann et al., 2012), but also of patient groups (O’Carroll and Papps, 2003; Ritter et al., 2004; Toplak et al., 2005, 2010; Dunn et al., 2006; Martino et al., 2007; van Holst et al., 2010; Upton et al., 2012).

The Iowa research group has identified the PDB phenomenon in clinical studies (Sevy et al., 2007; Johnson et al., 2008). Notably, the description for deck B in Bechara’s (2007) professional manual is different from the basic IGT assumption (Bechara et al., 1994). Descriptions for the decks in the manual are as follows:

**Deck A’**. Deck A’ is almost universally avoided by neurologically intact individuals. A high number of cards selected from this deck is strongly indicative of the presence of decision-making impairments in the examinee … **Deck B’**. A low number of cards selected from Deck B’ is strongly indicative of good or advantageous decision-making capacity on the part of the examinee. However, a high number of cards selected from this deck is less conclusive because the total number of selections from this deck in neurologically intact individuals can approach that of neurologically impaired patients…… (Bechara, 2007, p. 9)

Obviously, these are critical statements differentiating bad deck A from bad deck B. In addition, some dissimilarity can be observed between good deck C and good deck D. The number of cards selected from bad deck B needed to conclude a bad decision is obviously higher than the needed number of cards selected from bad deck A. This makes the judgment score in the clinical IGT version, that is [(C + D)−(A + B)], relatively looser than the judgment score in the original IGT, which is [(C + D)−(A + B) = 40] (see also Chiu et al., 2012). Therefore, we hypothesized that the categorization using the clinical IGT and original IGT versions would significantly differ.

On one hand, to validate inconsistencies between findings from the use of the professional norm published by Bechara (2007) and the basic assumption by Bechara et al. (1994), the present study employed the clinical IGT version as a research tool. The clinical IGT version was repeated three times (stages) with normal subjects to observe the extended validity, reliability, and the practice effect.

On the other hand, although some studies have suggested that factors, such as personality, negative affect (Buelow and Suhr, 2009; Hammers and Suhr, 2010), and executive functions (Gansler et al., 2011a,b), may considerably affect the performance of subjects and influence IGT validity, we nevertheless considered that the PDB phenomenon may provide alternative causality for the IGT validity problem.

Furthermore, we adopted not only traditional IGT analysis methods (four decks and five-block learning curves), but also followed some parts of the research method adopted by Hammers and Suhr (2010). We used the professional norm (criteria) in the published manual (Bechara, 2007) and also criteria based on basic assumption (Bechara et al., 1994) to categorize the performance of normal subjects and label good and bad decision makers. In short, we hypothesized that the critical problem of IGT validity lies in the PDB phenomenon, that is, most normal subjects – not just a few – would prefer the frequent gains offered by bad deck B. Specifically, we proposed that GLF may be the most critical factor in establishing the constructional validity and reliability of the clinical IGT version.

## Materials and Methods

### Participants

In the experiment, 72 subjects completed the clinical IGT version. The number of males (35) and females (37) was kept almost equal to eliminate the effects of gender-based differences. All subjects were recruited from the campus of Soochow University. Data were analyzed at the group level and reported anonymously.

### Task

The gain-loss structure of the clinical IGT version published by Bechara (2007) was used in this study (Table 2; see also Takano et al., 2010). Instructions for the IGT were provided to each subject. All subjects played a three-stage clinical IGT version (100 trials per stage) to determine their extended preference. At the start of Stages 2 and 3, subjects were informed that the internal rules of the game were the same as those in the preceding stage. The deck position was identical on the computer screen across three stages.

### Procedure

Subjects played the clinical IGT version thrice. At the start of the game, they were given the original introduction to IGT (Bechara et al., 1999, 2000; Bechara, 2007). Care was taken to ensure that all subjects understood how the game would be played on the computer. After Stages 1 and 2, participants were asked to play the game once more, and they were emphatically informed that the internal rules of the game in Stage 2 (Stage 3) were the same as those in Stage 1 (Stage 2). There have only a few minutes break between each two stages for subjects. The break allowed experimenters to reload the computer program and provide a short introduction for following stages.

### Data analysis

#### Four deck, net score, and learning curve analysis

The average number of cards selected, the net score [(C + D)−(A + B)] for five blocks (each block includes 20 trials), and the learning curve (depicted with five blocks) for each deck were used to describe findings between decks and infer the weights of factors (EV vs. GLF). In the general linear model, repeated measurements were made to evaluate the effect of two factors, namely, EV [(C + D) vs. (A + B)] and GLF [(A + C) vs. (B + D)]. In addition, one-way ANOVA and *post hoc* analysis were carried out to determine differences among decks (A, B, C, and D) and assess the effect of learning on the subjects. The exact difference between two decks in each stage was evaluated by paired sample *t*-test.

#### Reliability and practice effect test: comparison between two stages

Multivariate test was conducted to examine test-retest reliability and the practice effect between two stages. *Post hoc* analysis was employed for each deck to observe at which stage the learning effect becomes significant.

#### Comparison between the basic IGT assumption and professional norm

The present study utilized the basic assumption of Bechara et al. (1994) and the published professional norm (Bechara, 2007) to classify bad and good decision makers. The number of good and bad decision makers was statistically tested (χ^2^) in each criterion (assumption) (Bechara et al., 1994 vs. Bechara, 2007). If the Iowa group is right, results of the two categorization methods should be nearly equal. The detailed criteria for both assumptions are as follows.

##### Criteria based on the basic assumption

According to Bechara et al. (1994), normal decision makers choose the good decks. Therefore, in the standard IGT administration (100 trials), the criterion of chance-level selection should be 25 cards on each deck. Bad choice pattern is defined as over-selecting 25 cards on bad decks A and B or under-selecting 25 cards on good decks C and D.

##### Criteria based on the professional norm

According to the norm in the IGT manual published by Bechara (2007, p. 38), college students in the present study must be screened with the professional norm as follows: age = 18–39 years and education = 13–15 years. Bad choice pattern is defined as over-selecting 22 cards on bad deck A, over-selecting 38 cards on bad deck B, under-selecting 13 cards on good deck C, or under-selecting 20 cards on good deck D.

We classified normal subjects into four types based on their choice patterns in relation to bad decks (L_A/L_B = low deck A and low deck B; L_A/H_B = low deck A and high deck B; H_A/L_B = high deck A and low deck B; and H_A/H_B = high deck A and high deck B). In addition, we classified normal subjects into four types based on their choice patterns in relation to the good decks (L_C/L_D = low deck C and low deck D; L_C/H_D = low deck C and high deck D; H_C/L_D = high deck C and low deck D; and H_C/H_D = high deck C and high deck D).

## Results

### Four decks analysis

**Figure 1:**
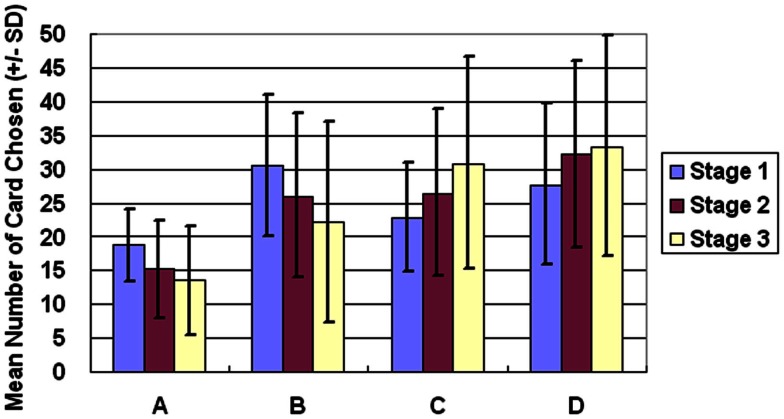
**Average number of cards chosen in each stage**. The average number of cards selected in the three stages demonstrates that the subject significantly prefers bad deck B to bad deck A in all three stages. Notably, normal decision makers significantly favor good decks C and D in Stage 3.

The general linear model revealed that GLF (decks B and D vs. decks A and C) – rather than EV (decks C and D vs. decks A and B) – dominated the choice behavior of subjects in Stage 1. The number of trials was the same as in the standard administration of the clinical IGT version. However, in Stage 2, the two factors, EV and GLF, equally influenced choice behavior. In Stage 3, EV overrode GLF in influencing choice behavior (Figure 1). Table 3 presents detailed statistics and the effects of the interaction between the two factors. Regression analysis (R-square) verified the above observation across the three stages (see η^2^ in Table 3).

**Table 3:** **The statistics of general linear model across three stages**.

Stage	Effect	*F*	Hypothesis df	Error df	*p*-Value	Partial η^2^
1	EV	0.36	1	71	0.55	0.01
	GLF	45.17	1	71	0.00**	0.39
	EV*GLF	7.66	1	71	0.00**	0.10
2	EV	28.30	1	71	0.00**	0.29
	GLF	28.44	1	71	0.00**	0.29
	EV*GLF	2.73	1	71	0.10	0.04
3	EV	42.18	1	71	0.00**	0.37
	GLF	9.85	1	71	0.00**	0.12
	EV*GLF	3.06	1	71	0.09	0.04

**p < 0.01, *p < 0.05.

One-way ANOVA and *post hoc* analysis were employed to compare the average number of cards selected in each stage between each pair of decks (A vs. B vs. C vs. D). Significant differences between the decks were evident in all three stages [Stage 1: *F*(3, 284) = 22.78, *p* < 0.01; Stage 2: *F*(3, 284) = 26.70, *p* < 0.01; Stage 3: *F*(3, 284) = 29.31, *p* < 0.01]. *Post hoc* analysis indicated that, in each stage, significant differences existed between each pair of decks under most conditions (Table 4), but differences between decks B and D in Stage 1, between decks B and C in Stage 2, and between decks C and D in Stage 3 were not significant. Table 4 presents detailed statistics obtained from the *post hoc* analysis. Notably, bad deck B was significantly preferred to bad deck A in all three stages.

**Table 4:** **The statistics of *post hoc* analysis across three stages**.

Bonferroni correction	Stage 1		Stage 2		Stage 3	
Paired deck	Mean differences	*p*-Value	Mean differences	*p*-Value	Mean differences	*p*-Value
A–B	−11.83	0.00**	−10.93	0.00**	−8.67	0.00**
A–C	−4.29	0.04*	−11.31	0.00**	−17.38	0.00**
A–D	−9.10	0.00**	−17.04	0.00**	−19.85	0.00**
B–C	7.54	0.00**	−0.38	1.00	−8.71	0.00**
B–D	2.74	0.47	−6.11	0.01*	−11.18	0.00**
C–D	−4.81	0.01*	−5.74	0.02*	−2.47	1.00

**p < 0.01, *p < 0.05.

### Net score and learning curves analysis

**Figure 2:**
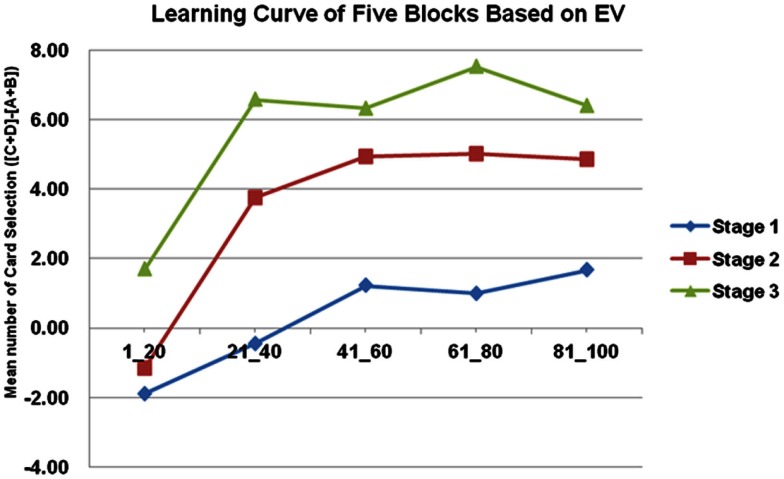
**Average number of cards in each block in each stage**. In the standard net score [(C + D)−(A + B)] in the clinical IGT version, the tendency to learn good EV is significant in each stage. The slopes of the learning curves increase rapidly in Stages 2 and 3. However, the standard clinical IGT version involves only 100 trials. The presentation of net scores has raised the question of whether normal decision makers truly avoid all of the bad EV cards and favor all of the good EV cards, and the question of why the difference between Block 1 and Block 5 in Stage 1 is only four cards [2−(−2)].

**Figure 3:**
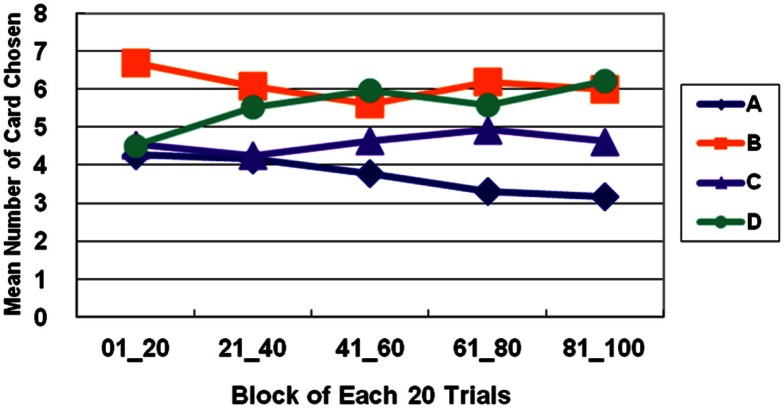
**Average number of cards selected in blocks in Stage 1**. The learning curves show that decks B and D (with high-frequency gain) are preferred to decks A and C (with low-frequency gain), even though bad deck B has a negative EV and good deck C has a positive EV. This observation verifies the PDB phenomenon and the theory that GLF dominates the choice behavior of decision makers.

**Figure 4:**
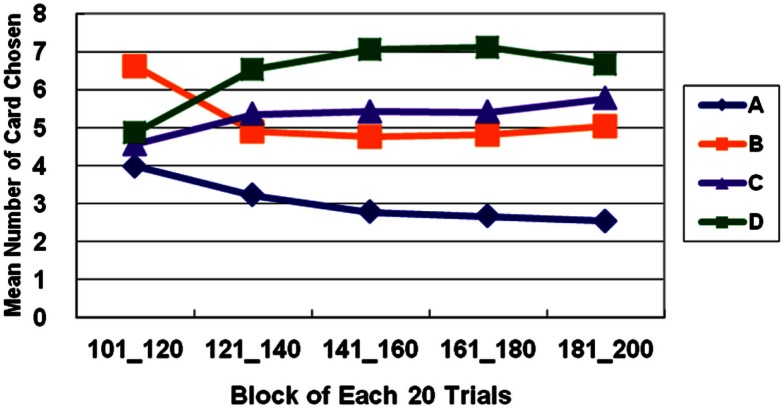
**Average number of cards selected in blocks in Stage 2**. In Stage 2, normal decision makers strongly prefer deck D (with high-frequency gain and positive EV). Bad deck B and good deck C are favored almost equally throughout most of the five blocks, and deck A with low-frequency gain and negative EV is consistently avoided.

**Figure 5:**
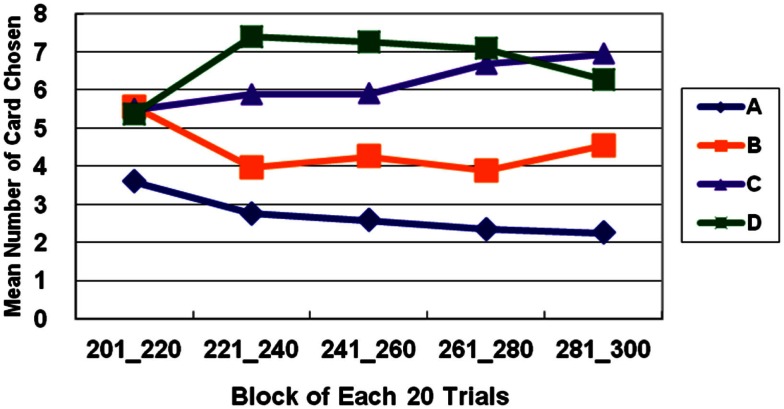
**Average number of cards selected in blocks in Stage 3**. In Stage 3, bad deck B is no longer preferred nearly as much as good deck C. The learning curves for the good and bad decks significantly diverge in Stage 3. Normal decision makers slowly come to favor decks C and D with positive EV and avoid decks A and B with negative EV.

The basic program of the clinical IGT version automatically generates a net score by subtracting the value of the bad deck from that of the good deck [(C + D)−(A + B)] for each block (20 trials/block). In each stage, these scores were plotted to generate a curve describing the performance of each subject. Thus, in the general linear model, repeated measurements of five blocks (Block 1–5) revealed that the subjects gradually learned to prefer the high EV choice (Figure 2) [Stage 1: *F*(4, 284) = 5.57, *p* < 0.01; Stage 2: *F*(4, 284) = 11.79, *p* < 0.01; Stage 3: *F*(4, 284) = 9.714, *p* < 0.01]. However, the details of the learning curve associated with each deck in each stage revealed that deck B was strongly preferred not only in Stage 1 but also in Stage 2 (Figures 3–5). A detailed paired *t*-test between each pair of decks in each block was conducted, and the subjects were found to prefer bad deck B to bad deck A. Notably, in Stage 1, bad deck B was chosen significantly more often than good deck C in each block (Figure 3). Even in Stage 2, bad deck B was nearly equal to that for good deck C (Figure 4) in the last four blocks. Table 5 presents detailed statistics for comparison. In summary, the curves demonstrate that the subjects preferred good decks C and D in most stages, but tended not to avoid bad deck B even in Stage 2. The PDB phenomenon was thus verified in the clinical IGT version.

**Table 5:** **The statistics of pair-*T* tests between each two decks in each block**.

Paired deck	Stage 1		Stage 2		Stage 3	
	T (df = 71)	*p*-Value	*T* (df = 71)	*p*-Value	*T* (df = 71)	*p*-Value
A1–B1	−5.14	0.00**	−5.33	0.00**	−4.43	0.00**
A2–B2	−4.69	0.00**	−4.28	0.00**	−3.40	0.00**
A3–B3	−3.86	0.00**	−4.16	0.00**	−3.61	0.00**
A4–B4	−5.44	0.00**	−4.35	0.00**	−3.26	0.00**
A5–B5	−4.98	0.00**	−4.52	0.00**	−3.92	0.00**
A1–C1	−1.08	0.28	−1.40	0.17	−3.34	0.00**
A2–C2	−0.34	0.73	−4.38	0.00**	−4.97	0.00**
A3–C3	−2.65	0.01*	−4.55	0.00**	−4.94	0.00**
A4–C4	−3.55	0.00**	−4.78	0.00**	−6.47	0.00**
A5–C5	−3.34	0.00**	−5.55	0.00**	−6.96	0.00**
A1–D1	−0.77	0.45	−1.79	0.08	−2.79	0.01*
A2–D2	−2.99	0.00**	−5.33	0.00**	−5.89	0.00**
A3–D3	−4.24	0.00**	−6.27	0.00**	−5.88	0.00**
A4–D4	−4.85	0.00**	−6.67	0.00**	−7.20	0.00**
A5–D5	−5.18	0.00**	−5.65	0.00**	−6.13	0.00**
B1–C1	4.26	0.00**	3.68	0.00**	0.10	0.92
B2–C2	4.45	0.00**	−0.75	0.45	−2.72	0.01*
B3–C3	1.87	0.07	−0.90	0.37	−1.98	0.05
B4–C4	1.78	0.08	−0.74	0.46	−3.25	0.00**
B5–C5	2.36	0.02*	−0.93	0.35	−2.51	0.02*
B1–D1	3.66	0.00**	2.54	0.01*	0.26	0.80
B2–D2	0.97	0.34	−2.24	0.03*	−3.92	0.00**
B3–D3	−0.63	0.53	−2.74	0.01*	−3.32	0.00**
B4–D4	0.82	0.42	−2.84	0.01*	−3.64	0.00**
B5–D5	−0.27	0.79	−1.76	0.08	−1.85	0.07
C1–D1	0.08	0.94	−0.53	0.60	0.18	0.86
C2–D2	−2.67	0.01*	−1.59	0.12	−1.47	0.15
C3–D3	−2.37	0.02*	−1.72	0.09	−1.28	0.20
C4–D4	−1.09	0.28	−1.93	0.06	−0.42	0.68
C5-D5	−2.27	0.03*	−0.96	0.34	0.69	0.49

**p < 0.01, *p < 0.05.

### Reliability and practice effect test: comparison between two stages

Significant differences were found among stages for all decks [deck A: *F*(2) = 10.13, *p* < 0.01; deck B: *F*(2) = 7.89, *p* < 0.01; deck C: *F*(2) = 7.29, *p* < 0.01; deck D: *F*(2) = 3.11, *p* < 0.05]. The learning effect by stages can be observed for bad decks A and B between Stages 1 and 2 and Stages 1 and 3, and for good decks C between Stages 1 and 3, and Stages 2 and 3, and D only between Stage 1 and 3 (Table 6).

**Table 6:** **The *post hoc* analysis for each deck between each two stages**.

Deck	Stage LSD correction	Mean difference	Hypothesis df	Error df	*P*-value
A	1–2	3.51	1	71	0.00**
	1–3	5.17	1	71	0.00**
	2–3	1.65	1	71	0.16
B	1–2	4.42	1	71	0.04*
	1–3	8.33	1	71	0.00**
	2–3	3.92	1	71	0.06
C	1–2	−3.50	1	71	0.09
	1–3	−7.92	1	71	0.00**
	2–3	−4.42	1	71	0.04*
D	1–2	−4.43	1	71	0.06
	1–3	−5.58	1	71	0.02*
	2–3	−1.15	1	71	0.63

**p < 0.01, *p < 0.05.

### Comparison between the basic assumption and professional norm

**Figure 6:**
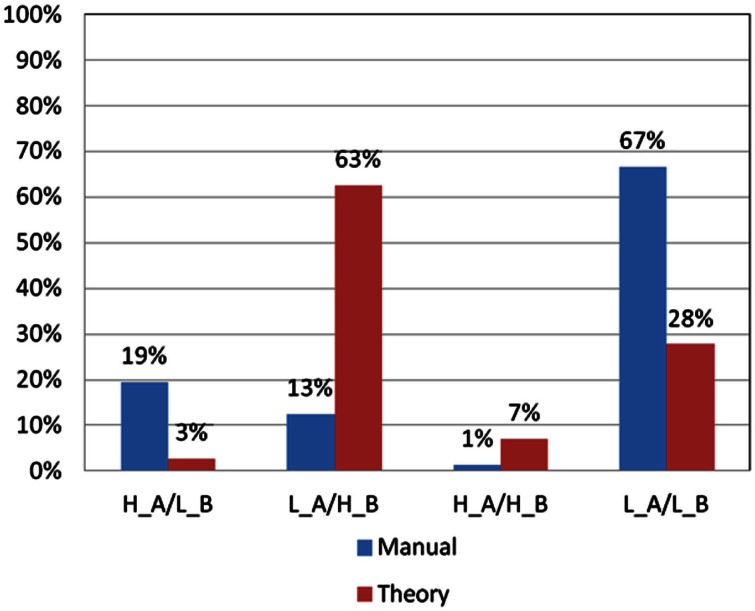
**Choice pattern comparison using different criteria for bad decks A and B**. According to criteria in the basic assumption and professional norm, the choice pattern of subjects for bad decks A and B can be categorized into four types (H_A/L_B: over-selecting deck A and under-selecting deck B; L_A/H_B: under-selecting deck A and over-selecting deck B; H_A/H_B: over-selecting deck A and over-selecting deck B; and L_A/L_B: under-selecting deck A and under-selecting deck B). Obviously, some significant differences can be observed in the L_A/H_B and L_A/L_B types when the two different criteria are used.

**Figure 7:**
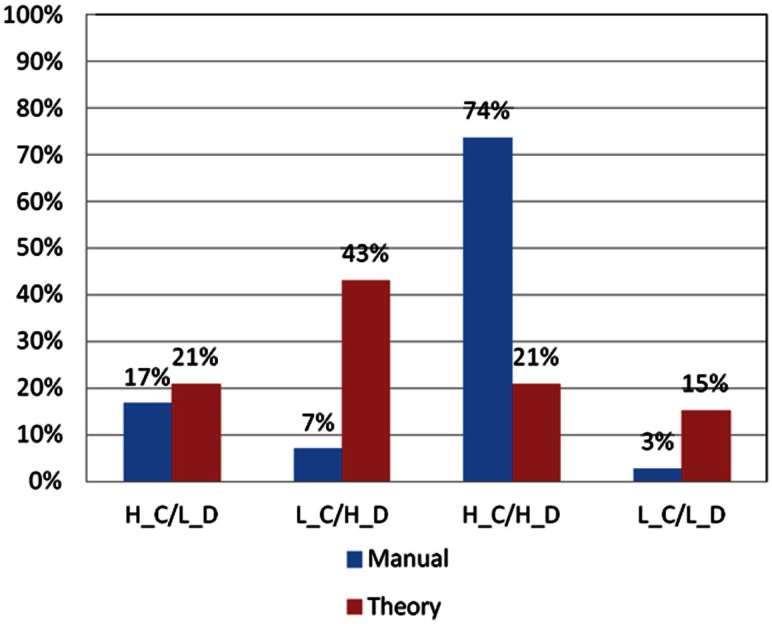
**Choice pattern comparison using different criteria for good decks C and D**. According to criteria in the basic assumption and professional norm, the choice pattern of subjects for good decks C and D can be categorized into four types (H_C/L_D: over-selecting deck C and under-selecting deck D; L_C/H_D: under-selecting deck C and over-selecting deck D; H_C/H_D: over-selecting deck C and over-selecting deck D; and L_C/L_D: under-selecting deck C and under-selecting deck D). Obviously, some significant differences can be observed in the L_C/H_D and H_C/H_D types when the two different criteria are used.

Significant differences were found when good and bad decision makers were classified based on the basic assumption and the professional norm (Figures 6 and 7).

As shown in Figure 6, the significant difference in the number of good and bad decision makers classified based on the basic assumption and the professional norm was primarily found in the L_A/H_B (low deck A and high deck B) group [χ*^2^*(2) = 29.25, *p* < 0.01] and L_A/L_B (low deck A and low deck B) group [χ*^2^*(2) = 41.33, *p* < 0.01]. In the L_A/H_B group, only nine subjects matched the criteria in the professional norm, whereas 45 subjects fitted the criteria in the basic assumption. Meanwhile, in the L_A/L_B group, 48 subjects matched the criteria in the professional norm, whereas only 20 subjects fitted the criteria in the basic assumption. No significant differences were found between the two criteria in terms of the choice patterns of subjects in the H_A/L_B group (high deck A and low deck B) and H_A/H_B group (high deck A and high deck B).

As shown in Figure 7, the significant difference in the number of subjects with good choice patterns classified based on the basic assumption and the professional norm was primarily found in the L_C/H_D (low deck C and high deck D) group [χ*^2^*(2) = 23.08, *p* < 0.01] and H_C/H_D (high deck C and high deck D) group [χ*^2^*(2) = 55.08, *p* < 0.01]. In the L_C/H_D group, only five subjects matched the criteria in the professional norm, whereas 31 subjects fitted the criteria in the basic assumption. In the H_C/H_D group, 53 subjects matched the criteria in the professional norm, whereas only 15 subjects fitted the criteria in the basic assumption. No significant differences were observed between classifications based on the basic assumption and the professional norm in terms of choice patterns in the L_C/L_D group (low deck C and low deck D) and H_C/L_D group (high deck C and low deck D).

## Discussion

In this study, the subjects focused on GLF in Stage 1 (Figures 1 and 2). They were more influenced by EV after Stage 2 (Figures 3–5), and the PDB phenomenon occurred not only in Stage 1 but also in Stage 2. This finding seriously affects the validity of the clinical IGT version in elucidating the decision behavior of neurologically impaired and psychiatric patients. Notably, even normal decision makers are more sensitive to GLF than to EV in the standard IGT (100 trials). Additionally, the degree of uncertainty experienced (more types of gain-loss value) by subjects regarding their decisions in the clinical IGT version (Bechara et al., 2000; Bechara, 2007) exceeds that in previous IGT version (Bechara et al., 1994).

The findings of this study in relation to the clinical IGT version are not isolated. Fernie and Tunney (2006) made similar findings in a two-stage IGT. They found that the number of people preferring deck B exceeds the number of those preferring the three other decks in Stage 1. Furthermore, some clinical investigations have noted no deficits in clinical groups (Wilder et al., 1998; Nielen et al., 2002).

A few studies have demonstrated the effect of GLF on decision making not only on control groups (Lin et al., 2007; Chiu et al., 2012), but also on clinical and experimental groups (Wilder et al., 1998; O’Carroll and Papps, 2003; Crone et al., 2004; Overman et al., 2004; Ritter et al., 2004; Bark et al., 2005; Rodriguez-Sanchez et al., 2005; Toplak et al., 2005; Caroselli et al., 2006; Fernie and Tunney, 2006; Martino et al., 2007; Fum et al., 2008; Fridberg et al., 2010). A review by Steingroever et al. (2013) involving eight datasets on control groups in clinical studies confirmed the PDB phenomenon and concluded that the use of IGT based on the basic assumption (EV) for testing the decision behavior of healthy subjects demands careful reconsideration.

Recently, Upton et al. (2012) utilized the IGT and SGT (Soochow Gambling Task, a modified version of IGT; Chiu et al., 2008) to compare the decision-making performance of normal subjects with that of opiate users. Results revealed that both groups were more sensitive to GLF than EV in both tasks. In addition, the opiate users performed worse than the normal controls in the IGT because of their inability to use GLF as a decision-making guide under uncertainty. This finding by Upton et al. (2012) is inconsistent with a previous finding by Bechara et al. (2002) concerning drug users. However, most of the Iowa group’s clinical studies (Bechara et al., 1998, 1999, 2000, 2001, 2002; Bechara and Damasio, 2002) presented their results in a net-score format [(C + D)−(A + B)], and this manner of presentation makes the detailed evaluation of both the PDB phenomenon and the number of selected cards from each deck invisible.

An increasing number of clinical researchers have noticed the PDB phenomenon, leading them to investigate the possibility of employing GLF in distinguishing clinical from control groups (Toplak et al., 2010; van Holst et al., 2010). Laboratories in Indiana University (Ahn et al., 2008) and Max-Plank Institute (Horstmann et al., 2012) have provided behavioral and modeling data related to IGT and have suggested that under uncertainty, the GLF is more important than EV.

Some pieces of evidence suggest that in Stage 3, subjects slowly start avoiding bad deck B and begin favoring good decks C and D. This result implies that GLF and EV dominate the choice behavior of subjects in different stages. In the initial stage (Stage 1), subjects adopt the gain-stay lose-shift strategy (Mitropoulos, 2003; Lin et al., 2007; Chiu et al., 2008; Cassotti et al., 2011) to overcome uncertainty. For instance, Chiu et al. (2008) demonstrated that subjects tend to remain on the decks while receiving the gains and shift their choice while they were receiving losses. On the other hand, they gradually lose their preference for immediate gain and fear of immediate loss in a stage with relative certainty (Stage 2 or 3). Hence, decision makers may use different strategies in short-run and long-run games. Normal subjects can learn to make the right decisions for long-term benefits if they can play the game with relative certainty regarding outcomes in the final stages. Briefly, in the clinical IGT version, GLF first, and later EV, can affect the decision patterns of normal subjects given more than 100 trials.

In the clinical IGT version, even normal decision makers require three stages (more than 200 trials) to reach standard performance (Bechara et al., 1994, 1999) as shown in Figures 1 and 5. If normal decision makers have a problem in reaching standard performance in the standard administration of IGT (100 trials), then the expected performance of clinical groups must be reconsidered. Moreover, the control group provides the baseline with which the results of the clinical group are compared. However, since control groups have consistently exhibited the PDB phenomenon in too many IGT-related investigations, neuropsychological assessment using the clinical IGT version should be reconsidered carefully (Chiu et al., 2012). Additionally, some studies have mentioned that the primary factor to be considered in distinguishing between clinical and control groups in the standard IGT administration may be GLF, not EV (Crone et al., 2003, 2005; Fernie and Tunney, 2006; Chiu and Lin, 2007; Fernie, 2007; Lin et al., 2007, 2008, 2009; Chiu et al., 2008; Fum et al., 2008; Stocco et al., 2009).

Actually, the Iowa group has provided some explanation for PDB in their published manual and mentioned that similar to the case of neurologically impaired patients, some normal subjects prefer to choose bad deck B (Bechara, 2007). Therefore, they suggested that verifying the decision ability of neurologically impaired patients based on their choice pattern for deck B is difficult. This statement clearly indicates that the preference for deck B is indistinguishable between normal subjects and neurologically impaired patients. Furthermore, if experimenters use the professional norm (enclosed in the manual) as reference for interpretation, they will find that even subjects who strongly prefer bad deck B cannot be categorized as myopic decision makers because the professional norm in relation to deck B selection has been broadened.

Classifications based on the basic IGT assumption may become inconsistent with those based on the professional norm. For instance, if experimenters use Table A2 (Bechara, 2007, p. 38), the professional norm for college students (age = 18–39 years and education = 13–15 years) as reference point, and some subjects choose cards from bad deck B 38 times (the chance-level is 25), these subjects will be classified as having normal performance (>16%). The interpretation for deck B according to the published manual is inconsistent with the basic IGT assumption but consistent with the present observation in this empirical study. However, to our knowledge, the deck B statement mentioned above has been used in turn to explain and further normalize the preference for deck B, which is incompatible with the basic IGT assumption (Kully-Martens et al., 2012). Therefore, the Iowa group should not only revise the interpretation in the manual but also directly modify the gain-loss structure of the IGT (Bechara, 2007).

### Reliability and practice effect test: Comparison between two stages

Buelow and Suhr (2009) stressed that the IGT lacks consideration of reliability and practice effect. The Iowa group completed the (test-retest) reliability examination and suggested that VMPFC patients still perform worse in IGT, but normal controls could improve their performance, even 1 day or 6 months later. The original statement by the Iowa group is as follows:

As a result of repeated testing, E.V.R.’s performance did not change, one way or the other, when tested one month after the first test, 24 h later, and for the fourth time, six months later. This pattern of impaired performance was also seen in other target subjects. On the contrary, the performance of normal controls improved over time. (Bechara et al., 1994, p. 13)

In the present study, normal subjects played the IGT three times consecutively and the net score [(C + D)−(A + B)] showed improvement across the three stages. The PDB phenomenon existed in the first two stages, which is quite inconsistent with the previous reliability report by the Iowa group. Additionally, the performance of the subjects for good vs. bad decks was diverse across the stages (Table 6; Figures 6 and 7), suggesting differences in the learning speed of subjects on each deck. The choice pattern changed between stages, but the PDB phenomenon existed in Stage 1, indicating that the reliability of the clinical IGT version was still yet to be established.

### Comparison between professional norm and basic assumption

The present study compared two criteria (professional norm vs. basic assumption) by filtering the choice patterns of 72 normal college students. Using the professional norm as a filter apparently underestimated the number of subjects choosing bad deck B by using a loose criterion (the criterion is largest among all decks, 38) in the professional manual (Bechara, 2007). We used the professional norm (B: 38) in filtering the choice patterns of subjects for bad deck B; only nine subjects over-selected bad deck B. Meanwhile, with the basic IGT assumption (B: 25), the number of subjects that over-selected bad deck B was increased to 45. This part is noticeably inconsistent with the basic IGT assumption (Figure 6).

Furthermore, in the L_A/L_B group, the number of subjects filtered by the criteria in the professional norm was lower than the number of subjects filtered by the criteria in the basic IGT assumption. Specifically, the criteria in the professional norm for bad deck B will make the interpretation become “*most healthy subjects could avoid bad decks A and B*.” Therefore, the criteria in the professional norm will become congruent with the basic IGT assumption. Nevertheless, most experimenters may not notice the criteria in the professional norm (Bechara, 2007) and that the basic assumption for deck B is completely different (Bechara et al., 1994). It is worth noting that few subjects preferred bad deck A in both criteria. This implies that bad deck A is a relatively more stable index than bad deck B in assessing normal subjects (Gansler et al., 2011a).

On the other hand, in terms of good choice patterns, two out of four categorical groups (L_C/H_D and H_C/H_D) demonstrated inconsistent classification results when the professional norm and basic assumption were used (Figure 7). Regardless of the criteria (professional norm or basic assumption) for filtering choice patterns, both methods showed a large number of subjects preferring good deck D. This implies that good deck D is a relatively more stable index than good deck C for establishing the constructional validity of IGT (Gansler et al., 2011a).

Based on the Iowa group’s serial studies, using the criteria in the professional norm or in the basic assumption to filter choice patterns for the same population should yield similar classification results. However, the present research illustrated that based on the criteria in the professional norm, 39 (54.2%) subjects were identified as foresighted decision makers (L_A/L_B/H_C & H_D). The identification criterion for good or bad choice patterns is looser when the professional norm is used than when the basic IGT assumption is used (Bechara et al., 1994). There were more foresighted decision makers than myopic decision makers.

Conversely, when the original viewpoint was used to classify the data, a very different categorization result emerged. A total of 8 (11.1%) subjects were identified as foresighted decision makers (L_A/L_B/H_C & H_D). Notably, most of the subjects were not identified as foresighted decision makers, namely, those who are unable to make a foresighted decision in the long run. This result is incongruent with the classification result based on the professional norm and is against the basic IGT assumption.

Not considering two factors, GLF and the practice effect, is risky for IGT validity. In short, the main purpose behind the design of the clinical (or computer-based) version by the Iowa group is to enlarge EV contrast (Table 2) between good and bad decks and further identify the myopic behavior of VMPFC patients (Bechara et al., 2000). However, in the present test, even normal subjects were found to have problems appreciating the high EV contrast between good and bad decks in the clinical (or computer-based) IGT version. At the same time, the sample size of subject group in this study was still limited. Also, the present study was lack of prescreening with the detailed neuropsychiatric criteria (e.g., Mini-Mental State Examination, Beck Depression Inventory, Beck Anxiety Inventory…). If the dataset for the extent of this study is used to refine the norm, these factors should be also carefully controlled for.

## Conclusion

Following several inconsistent studies on the PDB phenomenon, the present study involved a three-stage experiment to test the validity and reliability of the clinical IGT version (Bechara, 2007). The PDB phenomenon was observed in the clinical IGT version when administered in the standard fashion (with 100 trials). Nevertheless, decision makers may adopt an immediate perspective under uncertainty (Stage 1) but make long-term deliberations under relative certainty (Stage 3). Consequently, this study suggests that the present format of the IGT is not only invalid for determining the choice patterns of normal decision makers, but also invalid for the clinical assessment of neuropsychological patients. GLF should be reconsidered in the future revision of the clinical IGT version.

## Author Contributions

Ching-Hung Lin, Tzu-Jiun Song, and Yao-Chu Chiu conceived and designed the experiment. Ying-Ying Chen and Tzu-Jiun Song recruited the volunteers and performed the experiment. Tzu-Jiun Song, Ying-Ying Chen, and Ching-Hung Lin contributed to the data analysis and result depiction. Ching-Hung Lin, Tzu-Jiun Song, and Yao-Chu Chiu interpreted the data and wrote the preliminary draft. We-Kang Lee provided some critical inputs regarding references and detailed the discussion of the clinical IGT version. Yao-Chu Chiu finalized the manuscript with input from all the authors.

## Conflict of Interest Statement

The authors declare that the research was conducted in the absence of any commercial or financial relationships that could be construed as a potential conflict of interest.
